# Mapping intervention components from a randomized controlled trial to scale-up of an early life nutrition and movement intervention: The INFANT program

**DOI:** 10.3389/fpubh.2022.1026856

**Published:** 2023-01-13

**Authors:** Sarah Marshall, Brittany J. Johnson, Kylie D. Hesketh, Karen J. Campbell, Kylie Fraser, Penelope Love, Elizabeth Denney-Wilson, Jo Salmon, Zoe McCallum, Rachel Laws

**Affiliations:** ^1^Institute for Physical Activity and Nutrition, School of Exercise and Nutrition Science, Deakin University, Geelong, VIC, Australia; ^2^Transforming Obesity Prevention in CHILDren (TOPCHILD) Collaboration, Sydney, NSW, Australia; ^3^Caring Futures Institute, College of Nursing and Health Sciences, Flinders University, Bedford Park, SA, Australia; ^4^NHMRC Centre of Research Excellence in Translating Early Prevention of Obesity in Childhood (EPOCH-Translate CRE), Sydney, NSW, Australia; ^5^Sydney Nursing School, Faculty of Medicine and Health, University of Sydney and Sydney Local Health District, Sydney, NSW, Australia; ^6^Department of Paediatrics, Melbourne Medical School, University of Melbourne, Melbourne, VIC, Australia

**Keywords:** behavioral intervention, behavior change techniques (BCTs), early childhood, dietary, physical activity, sedentary behavior, parents and caregivers

## Abstract

**Introduction:**

Early life parent-focused interventions can effectively improve infant and child nutrition and movement (physical activity and sedentary behavior) as well as parents' health behaviors. Scale-up of such interventions to real-world settings is essential for population-wide benefits. When progressing to scale-up, intervention components may be modified to reflect contextual factors and promote feasibility of scale-up. The INFANT program, an efficacious early life nutrition and movement behavioral intervention began as a randomized controlled trial (RCT), was modified after a small-scale translation, and is currently being scaled-up in Victoria, Australia. This study mapped and compared discrete intervention components of both the original RCT and the scaled-up version of INFANT to examine modifications for scaling up.

**Methods:**

Discrete intervention components, specifically the target behaviors (child-related and parent-related behaviors), delivery features and behavior change techniques (BCTs) from the RCT and the scaled-up program were coded and mapped using established frameworks and taxonomies. Publications and unpublished materials (e.g., facilitator notes, handouts, videos, app) were coded. Coding was performed independently in duplicate, with final coding validated in a meeting with interventionists. Interventionists reported the rationale for modifications made.

**Results:**

The INFANT RCT and scaled-up version targeted the same obesity prevention-related nutrition and movement behaviors. Key modified delivery features at scale-up included reduced number of sessions, a broader range of professionals facilitating groups, the addition of a mobile app for parents replacing hard-copy materials and tangible tools (e.g., pedometers), and broadening of content (e.g., early feeding, updated 24-h movement guidelines). BCTs used across the RCT and scale-up sessions were unchanged. However, the BCTs identified in the between-session support materials were almost double for the scale-up compared with the RCT, primarily due to the reduced number of sessions and the app's capacity to include more content.

**Conclusions:**

INFANT is one of few early life nutrition and movement behavioral interventions being delivered at scale. With INFANT as an example, this study provides critical understanding about what and why intervention components were altered as the RCT was scaled-up. Unpacking these intervention modifications provides important insights for scale-up feasibility, outcome effects, and how to optimize implementation strategies for population-level benefits.

## Introduction

Establishing optimal nutrition and movement behaviors (physical activity and sedentary behavior) in early life is critical for achieving health and wellbeing benefits that track into adulthood, including preventing overweight and obesity (1, 2). Family and parental-child influences are important for establishing healthy early life nutrition and movement behaviors (2–5). Early childhood family-based behavior change interventions are an important avenue for supporting optimal nutrition and movement behaviors; such interventions have been shown to reduce obesity risk behaviors in children aged 0–5 years (6–8). While there is evidence about what works in controlled research studies, few interventions progress to implementation at scale (9, 10).

Scale-up refers to the expansion of health interventions or innovations tested under research conditions to reach more people and achieve sustained benefits (11). Scale-up is essential for population-level reach and impact, providing opportunity to contribute to widespread improvements in children's health and wellbeing. However, three recent systematic reviews (12–14) of scaled-up obesity, physical activity and nutrition interventions targeting varied populations, report that most scaled-up interventions only achieve 50–75% of original trial effect size. This scale-up “penalty” may reflect the modifications that are made to an intervention when it moves from randomized controlled trial (RCT) to scale-up to make it more feasible to deliver within a given practice and policy context. Scaled-up intervention modifications may include changes to the target audience, target behaviors and delivery features (e.g., who delivers the intervention, how and where it is delivered and at what dose). These modifications may impact the intervention's effective components, which may explain the drop in effect size from RCT to scaled-up delivery. Further research is needed to better understand what and why modifications occur during the scale-up process (15), and how this impacts the effective components used between trial and scaled-up interventions.

Behavior change interventions for preventing childhood obesity are complex and contain multiple components. It is vital to have methods for describing intervention components with consistency, transferability, and specificity (16, 17). Intervention components can be described as the target behaviors, delivery features and Behavior Change Techniques (BCTs). A BCT is “an observable, replicable, and irreducible component of an intervention designed to change behavior and a postulated active ingredient within the intervention” (17). Frameworks, such as BCT taxonomies and intervention reporting templates, provide a standardized approach for describing, identifying, and specifying components of complex behavior change interventions. Deconstructing interventions into their components enables consistent and complete reporting, replicability and an investigation of which components are most likely to contribute effective behavior change.

Describing intervention components can contribute to developing, enhancing, and understanding effective behavior change interventions. Building on previous work examining BCTs used in early childhood obesity prevention interventions in Australia and New Zealand (18), a comprehensive global review is underway to characterize the effective components of early childhood obesity prevention interventions to identify the components used to target infant nutrition, sleep and movement behaviors (19, 20). Other previous systematic reviews have identified BCTs associated with effective interventions related to childhood obesity prevention (21–23). However, all studies and reviews to-date focus on controlled trials; little is known about intervention components of early obesity prevention interventions at scale.

The INFANT (INfant Feeding, Active play and NuTrition) program is the first evidence-based early childhood intervention targeting parents and caregivers aiming to improve child nutrition and movement behaviors to be scaled-up in Australia. INFANT was developed with input from child health experts, health professionals and parents and, in 2008, was delivered as a randomized controlled trial (RCT) with 542 families in Victoria, Australia (24). In the RCT, INFANT consisted of six group sessions with mothers, held over the first 18 months of their child's life, led by a dietitian. The intervention showed positive maternal and child outcomes under controlled conditions, specifically, improved maternal dietary patterns, self-efficacy and knowledge, and improved child diet (fewer sweet snacks and improved dietary quality) and reduced child sedentary time (less television viewing) (25–27). Positive intervention benefits for several targeted child behaviors were sustained up to school age. Children in the intervention group had increased fruit, vegetable and water intake and fewer sweet snacks at 2 years post-intervention (child aged 3.5 years), and fewer sweetened drinks and fewer sweet snacks at 3.5 years post-intervention (child aged 5 years) (28). Further to this, at both follow-up timepoints, improved maternal television viewing knowledge was maintained and associated with less television viewing time among their children (29).

In 2012, INFANT was delivered as a small-scale translation trial, where uptake was high and provided proof of concept for implementation at scale (30, 31). INFANT is currently being scaled-up across Victoria, Australia and evaluated as a hybrid implementation-effectiveness trial in partnership with ten policy, practice, and research partners (32). Implementation support is enhanced through funding from the Victorian Department of Health and evaluation was funded by a National Health and Medical Research Council partnership grant (2019–2024, GNT1161223). The evaluation is in progress and aims to assess real-world implementation, effectiveness and cost-effectiveness of INFANT when delivered at scale.

The aim of this study is to examine how the intervention components (target behaviors, delivery features and BCTs) of INFANT changed from RCT to scale-up, and to explore factors that influenced these modifications. This will provide important new insights into how scale-up may impact the components of behavioral interventions to then better understand, and potentially mitigate, the frequently observed scale-up penalty.

## Materials and methods

### Study design

In May-July 2022, we undertook systematic mapping and analysis to compare the intervention components, namely target behaviors, delivery features, and BCTs of the INFANT RCT and the INFANT scale-up. This study leverages procedures and intervention coding conducted as part of the Transforming Obesity Prevention for CHILDren (TOPCHILD) Collaboration, specifically coding of the INFANT RCT delivery features, child-related target behaviors and corresponding BCTs (20). Recommendations for best practice application of BCT taxonomies in childhood obesity prevention were applied in the current study methods (e.g., duplicate independent coders) and reporting (e.g., detailed methods, referring to BCTs with the taxonomy number and label) (33) as described below.

### Target population, target behaviors, and delivery features

Parents are the target population of the INFANT program, with parental behaviors targeting change in children's health behaviors (i.e., child-related behaviors) and parents' own health behaviors (i.e., parent-related behaviors). The target population and target behaviors used in the INFANT program were identified from published papers and discussion with INFANT program designers (KJC, KDH) and related to parental feeding practices, dietary intake, physical activity, sedentary behavior of both children and parents, as well as parental wellbeing.

Delivery features were coded using pre-specified categories according to the Template for Intervention Description and Replication (TIDieR) checklist for reporting interventions (16). The TIDieR checklist consists of 12 items to aid consistent reporting of interventions. Items describe delivery of the intervention and include intervention name, the rationale or theory underpinning the intervention, intervention materials, procedures, delivery agents, mode of delivery setting/ location of intervention delivery, intervention dose, and any tailoring of the intervention or modifications made during delivery along with plans for maintaining fidelity and actual fidelity (16). The TIDieR checklist is particularly useful for reporting RCTs, yet has scope for enhancements for reporting intervention implementation and scale-up (34). To tailor the TIDieR checklist for this study, we added a column to describe the rationale for changes and an additional item of ‘context' to enable description of the environment in which the intervention was delivered, acknowledging that context is central to implementation (35).

### Behavior change technique coding and synthesis

We used the standardized reporting BCT Taxonomy v1 (BCTTv1) from Michie and colleagues (17). This taxonomy consists of 93 techniques, hierarchically clustered within 16 categories, with labels, definitions, and examples. The TOPCHILD Collaboration BCT codebook, with examples of the application of each technique to early childhood obesity prevention interventions, was used to assist with coding (20). BCTs were coded separately for the behaviors targeted across two populations (child-related health behaviors and parent-related behaviors), and for intervention sessions, videos and between-session materials across both the RCT and scale-up versions of the intervention.

All coders (BJJ, SP, SM, KF) completed the open-access University College London BCTTv1 online training (36). BJJ previously undertook further specialized training at University College London Centre for Behavior Change, completing the Behavior Change – Principles and Practice course. BJJ and SP have prior experience applying the BCTTv1 to childhood obesity prevention interventions. SM and KF were less experienced in applying BCTTv1 and consulted regularly with BJJ as an experienced coder. All coders had at least an undergraduate degree in a health-related field. Coders SP and KF had limited previous knowledge of the INFANT intervention; BJJ and SM were familiar with the INFANT RCT from previous projects, and SM was familiar with the INFANT scale-up through coordinating the implementation-effectiveness research project. No coders developed or delivered the RCT or scale-up intervention.

Published and unpublished intervention materials from the RCT and scale-up (such as session facilitator guides, newsletters, handouts, videos, app) were coded (detailed in the Supplementary material). For the RCT, all intervention materials were coded line-by line. For the scale-up, all intervention materials were coded line-by-line except for the mobile phone app for parents. Given the volume of content in the app, SM and KF independently selected a random sample of app content to code across all app features (i.e., articles, activities, push notifications and forum) until several examples were evidenced for the BCT or no evidence of the BCT was present. BJJ and SP independently coded the RCT materials. SM and KF independently coded the scale-up materials. BJJ checked all final coding and was part of the consensus process to discuss areas of ambiguity. An Excel template for “BCT present,” “source material,” and “direct excepts” was used by coders. BCTs were coded as yes/present or no/not present based on the BCT definitions, the codebook and coders' judgements to categorize intervention content. If there was coder uncertainty due to insufficient evidence, the BCT was coded as maybe/unsure. Coders met to discuss and agree upon discrepancies separately for the RCT, then the scale-up. For consistency, SM, BJJ and KF were involved in consensus meetings for both the RCT and scale-up. At this point, BCTs could remain coded as “maybe.”

The level of agreement between independent coders' BCT coding was assessed using prevalence-adjusted and bias-adjusted kappa (PABAK) to account for the high prevalence of negative agreement between coders (i.e., when both agree that a BCT is not present) (37). PABAK agreement values above 0.81 are classified as “excellent/almost perfect,” between 0.61 to 0.80 are classified as “substantial agreement,” 0.41 to 0.60 are “moderate” and below 0.4 are classified as “fair” agreement (38).

### Validation meetings

Coding validation with lead interventionists is not commonly undertaken when retrospectively coding BCTs, but is an important step for verification of intervention components that may be unclear from the descriptions in publications and available unpublished intervention materials. A validation process is currently being developed and tested as part of the TOPCHILD Collaboration to clarify any BCTs coded as “maybe” as well as confirm target behaviors and delivery features coded to ensure intervention coding aligned with intervention intent (20); the pilot validation methods were applied in the current study. This involved meeting online to discuss and review the coding with the lead interventionists. BJJ reviewed the RCT coding with leads investigators of INFANT RCT (KDH and KJC). SM, BJJ, and KF reviewed the scale-up coding with lead investigators of INFANT scale-up (RL, PL, KJC, KDH, EDW). BCTs were narratively contrasted and compared between the RCT and the scale-up and discussed with the interventionists to explore the rationale behind any intentional changes made to the intervention during the scale-up process over the 12-year period.

## Results

### Changes to target population, target behaviors, and delivery features from RCT to scale-up

Enhancements to the INFANT intervention from efficacy testing to scale-up were informed by RCT and small-scale implementation experiences (32). This included end-user evaluation studies with both practitioners (31) and parents (39–41). Decisions regarding planned adjustments for scale-up were made in close consultation with an implementation advisory committee consisting of interventionists and key practice and policy stakeholders. The committee was established prior to INFANT scale-up commencement and continues to meet several times per year to inform implementation and scale-up strategies.

Adjustments were made to the eligible target population for the INFANT intervention from the RCT to the scale-up. In the RCT, the intervention participants were first-time parents of children from 3 months of age. For the scale-up, the target population was expanded to any parents of children (as an option, determined by local sites) with intervention commencement from birth. Expanding beyond first-time parents and caregivers was to increase reach and potential benefits as well as fit adjusted local delivery set-up. Earlier intervention commencement was to enable inclusion of anticipatory breastfeeding information via the mobile app before the first group session.

Target behaviors related to children and parents for the RCT and scaled-up version of the INFANT intervention were largely unchanged (Table 1). Most child-related behaviors related to the primary intervention outcomes in both the RCT and scale-up. The exception was the addition of some infant feeding behaviors in the scale-up version (i.e., promoting breastfeeding, appropriate formula feeding and responsive milk feeding). The RCT was designed to commence at 3 months of age to align with the timing of established first-time parent groups led by community Maternal and Child Health Nurses as part of the free universal healthcare system in Victoria, Australia. In discussion with the interventionists, the advice from practitioners and experts when designing the RCT intervention was that delivering breastfeeding content at around 3 months of age (when the RCT commenced) may isolate or disengage some participants, as feeding mode is likely to be already determined. The addition of the app in the scale-up, allowed the intervention to begin from birth with potential to influence milk feeding decisions and assist with anticipatory guidance for overcoming challenges. Content about breast, formula and mixed feeding, including a new key message “feeding is a learning curve,” was added to the app to support parents before attending sessions and added as a new section on responsive milk feeding in first INFANT group session (held at around 3 months or age). This was informed by a feasibility study of an earlier version of the app that showed the value of providing support to parents from birth on breastfeeding and optimal formula feeding (if not breastfeeding) (42).

**Table 1:** Child and parent-focused behaviors targeted in the INFANT RCT and scale-up.

**Behavior cluster**	Target behaviors^*^
**Child-related**	
Infant feeding practices	• ^∧∧^Promoting and/or continuing breastfeeding, including exclusive breastfeeding to 6 months of age • ^∧∧^Feeding formula appropriately, if relevant (e.g., choice of formula preparation and bottle feeding) • ^∧∧^Responsive milk feeding (i.e., feeding in response to the infant's hunger/satiety cues) • Delaying introduction of solid foods (complementary feeding) until 6 months of age
Food provision and dietary intake	• Providing appropriate types of foods (e.g., vegetables, meat and alternatives, fruits, whole grains, dairy) • Providing age-appropriate portions of each food group (i.e., portion sizes) • Limiting provision of certain foods and drinks (e.g., energy-dense, nutrient poor foods, sugar-sweetened beverages) • Offering foods repeatedly that have previously been rejected • Offering foods and drinks in response to the infant's hunger/satiety cues [Responsive feeding] (e.g., letting the infant decide how much they eat, not pressuring to eat) • Avoiding use of food to control (or reward) the infant's emotions, behavior, or consumption of other foods • Providing regular meal routines (including eating together which models eating, limiting distractions)
Physical activity	• Placing infant on their stomach for prone play (“tummy time”) • Promoting age-appropriate physical activity such as active play, outdoor play, activities relating to fundamental movement skills • Providing toys that promote movement such as balls and toys on wheels • Providing a safe space for unrestricted play
Sedentary behaviors	• Limiting the amount of time that the infant is restrained (e.g., prams/strollers, high-chairs, strapped on a caregiver's back) • Limiting the amount of time that the infant is exposed to screens (e.g., television, mobile devices) • Providing alternatives to screen time • Modeling screen behaviors
**Parent-related**	
Dietary intake	• Increasing consumption of vegetables and fruit, drinking water • Limiting certain foods and drinks (e.g., energy-dense, nutrient poor foods, sugar-sweetened beverages) • Promoting family meals
Physical activity	• Increasing physical activity (e.g., walking, post-natal exercises)
Sedentary behaviors	• Reducing screen time • No television or screens at mealtimes
Wellbeing	• Encouraging and facilitating social connectedness • Promoting general health and wellbeing (e.g., self-care, seek support from friends and family) • Increasing parenting confidence

* Adapted from the TOPCHILD Collaboration target behavior clusters and example behaviors (20).

∧∧ Denotes behaviors added for the scale-up only.

Delivery features related to children and parents for the RCT and scaled-up version of the INFANT intervention are described in Table 2 along with the rationale for these changes. Changes to delivery features were informed by small scale translation studies with end users (both parents and practitioners) (30, 31, 41) and were primarily enacted to improve scalability and in response to temporal changes such as mothers' earlier return to work, availability and use of mobile phone apps and changes in preferences for online information.

**Table 2:** Intervention delivery features of INFANT RCT and scale-up, including rationale for changes.

Delivery feature category (TIDieR item^*^)	**RCT**	**Scale-up**	**Rationale for changes**
Item 1. Brief name *Provide the name or a phrase that describes the intervention*	InFANT: The Melbourne Infant Feeding, Activity and Nutrition Trial (InFANT) Program - a community-based, cluster-randomized controlled trial of an early intervention promoting healthy eating and active play, and in turn, healthy growth from the start of life.	INFANT: INfant Feeding, Activity and NutriTion (INFANT) - an early intervention promoting healthy eating and active play, and in turn, healthy growth from the start of life.	Updated intervention acronym for the scale-up to remove reference to the trial.
Item 2. Why *Describe any rationale, theory, or goal of the elements essential to the intervention*	Anticipatory guidance framework Social Cognitive Theory Parenting support theory	Anticipatory guidance framework Social Cognitive Theory Parenting support theory COM-B model of behavior	The underpinning theories for the sessions were unchanged. The COM-B model of behavior change (published in 2011, after the RCT) was used to inform the development of the app.
Item 3. What (materials) *Describe any physical or informational materials used in the intervention, including those provided to participants or used in intervention delivery or in training of intervention providers*	Session delivery - Facilitator session guides Resources provided to participants - Parent INFANT session handout (one per session) - Parent INFANT topic-specific handouts - Additional brochures from reputable sources (e.g., Australian Dietary Guidelines) - DVD with session videos - Between session newsletters - Tangible tools – ball, active play storybook, water bottle, shopping bag, pedometer Materials for training intervention providers - Face to face interactive group training sessions - Facilitator session guides used as training guide - Provider-trainer group emails between training sessions for support and troubleshooting challenges	Session delivery - Facilitator session guides Resources provided to participants - Parent INFANT session summary with reference to relevant app sections (optional printout) - Videos *via* internet/app - Mobile app Materials for training intervention providers - Comprehensive online training *via* a learning management system - Implementation guide	Addition of app due to advances in technology and parental preference for online supplementary information. Removal of tangible tools from the RCT due to cost and feasibility at scale. Videos were reduced in length due to generational preference for briefer visual content online.
Item 4. What (procedures) *Describe each of the procedures, activities, and/or processes used in the intervention, including any enabling or support activities*	Facilitated group discussions, including watching the videos. Peer support Exploration of barriers Interactive activities (e.g., tummy time with babies together) Reference to and promotion of the DVDs and other take-home materials during the sessions.	Facilitated group discussions, including watching the videos Peer support Exploration of barriers Interactive activities (e.g., tummy time with babies together) App push notifications, activities (self-completed quizzes for personalized feedback) and parent forum Promotion of the app to parents from their infant's birth. Reference to and promotion of the app during sessions.	Reduced number of activities in the sessions, allowing for reduced session time (2 to 1.5 h) due to limited workforce capacity at scale and the inclusion of some of the activities in the app.
Item 5. Who provided *For each category of intervention provider (e.g., psychologist, nursing assistant), describe their expertise, background and any specific training given*	Intervention provider: Research dietitians employed by research team. Training of intervention provider: 2-h face-to-face training meetings prior to each round of INFANT sessions (6 in total), facilitated by lead researchers/ interventionists.	Intervention provider: Delivered as part of routine practice by practitioners such as dietitians, maternal and child health nurses, health promotion officers, midwives, other parenting support or allied health workers. Training of intervention provider: 8–10-h online training course offered over a 4-6 week period (2–4 times per year) facilitated by lead interventionists and implementation experts. Annual 1–2-h online refresher training.	Delivery agent expanded to offer flexibility according to organization and staff capacity given that no additional funding was provided for delivery. Evidence from small scale translation suggested that a wider group of health professionals could deliver the intervention once trained. Online training was offered to address challenges faced by facilitators attending face-to-face training and logistics of waiting for a cohort of participants. This also allowed broader reach and reduced cost of the training.
Item 6. How *Describe the modes of delivery (e.g., face-to-face or by some other mechanism, such as internet or telephone) of the intervention and whether it was provided individually or in a group*	Face-to-face group sessions DVD and printed material provided in sessions Printed newsletters sent via mail between sessions	Face-to-face group sessions Mobile phone app including notifications	Addition of app due to availability of this new technology facilitated the provision of all information in one place, at one time, able to be updated with changes in knowledge/guidelines, convenience for parents and facilitators and cost effective over longer term.
Item 7. Where *Describe the type(s) of location(s) where the intervention occurred, including any necessary infrastructure or relevant features*	Community facilities close to where first-time parent group sessions were held (e.g., Maternal and Child Health centers, libraries, community halls). Sessions were delivered within existing first-time parent groups led by community Maternal and Child Health Nurses as part of the free universal healthcare system in Victoria, Australia. INFANT sessions started with the group directly after the nurses' content concluded/ when parents took over their own management of the groups.	Community facilities (e.g., Maternal and Child Health centers, community health organization group rooms, libraries). Sessions not limited to existing first-time parent groups. Organizations have the option to adopt this approach, but it is not essential. Groups may be constructed for the purpose of delivery or embedded into existing groups.	In the scale-up, specific venue choice to be determined by the organization delivering the program. To allow flexibility for implementation at scale, local organizations determined referral pathways and program set-up.
Item 8. When and how much *Describe the number of times the intervention was delivered and over what period of time including the number of sessions, their schedule, and their duration, intensity or dose*	Total intervention period: 15 months. 6x 2 h group sessions at 3, 6, 9, 12, 15, 18 months of age. 5x Newsletters sent between sessions	Total intervention period: 18 months. 4x 1.5 h group sessions at 3, 6, 9, 12 months of age. Additional support *via* app, including push notifications and discussion forum between birth and 18 months.	Parents' earlier return to work (between 9 and 12 months as opposed to 15–18 months in the original trial) necessitated condensing content into 4 rather than 6 sessions concluding at 12 months but with app support to 18 months. Addition of the app also in response to greater availability of online information and advances in technology.
Item 9. Tailoring *If the intervention was planned to be personalized, titrated or adapted for individual participants then describe what, why, when, and how*	Group discussions were tailored to participants' preferences, concerns, or situations	Group discussions were tailored to participants' preferences, concerns, or situations. The app push notifications tailored according to participant's feeding mode (breast, formula or mixed feeding) and child age and stage of development.	Formative research indicated importance of tailored app push notifications and the app delivery format allowed for this. Technology advances enabled addition of this feature.
Item 10. Modifications *If the intervention was modified during the course of the study, describe the changes (what, why, when, and how)*.	Removal of text message component from trial protocol due to funding constraints.	The potential for local modifications are described in the online INFANT implementation training (e.g., venue, recruitment, facilitators, partner organizations). Local implementation and evaluation data collection is currently underway; therefore, modifications made by local areas are currently unknown.	Modifications to allow flexibility for implementation at scale, including local contexts and community characteristics.
Item 11 and 12. How well (planned or actual) *Planned:* describe how and by whom, and if any strategies were used to maintain or improve fidelity, describe them Actual: *describe the extent to which the intervention was delivered as planned*.	Planned: - Standardized session outline for facilitators to improve fidelity - Between-session newsletters sent to participants to remind of key messages and promote adherence Actual: - Program fidelity was audited via checklists by researchers attending but not delivering the intervention. - 68% of participants attended 4 or more of the 6 sessions	Planned: - Standardized session outline for facilitators to improve fidelity - Data collection planned for monitoring fidelity includes: a) undertaking fidelity checklists from a subset of implementing sites b) facilitator reporting of delivery of intervention in 12 month post-training survey Actual: Program implementation and data collection in progress; therefore, fidelity is currently uncertain.	Fidelity measures adjusted according to scale-up evaluation. Fidelity outcomes not yet available for the scale-up.
Additional item. Context *Not a TIDieR item. Added to capture additional contextual factors such as funding and broader environment*	Timeframe: RCT conducted from 2008 to 2010 Lead organization: Deakin University, research interventionists Environment: Occurred prior to policy/programs emphasis on pregnancy or early life period, most interventions started from school-age Funding: The National Health and Medical Research Council Grant GNT425801.	Timeframe: Scale-up across Victoria commenced in 2020 and is currently underway Lead organization: Overseen by Deakin University research interventionists, led by local government areas and services Environment: Occurring in the context of COVID-19 pandemic and Victoria's extensive lockdown periods Funding: Funding to enhance implementation provided by Victorian Department of Health (supports training at no cost to practitioners, seed funding for establishing the program, and implementation support). No additional funding for delivery for local organizations is currently provided. An evaluation of the scale-up is being funded by a 5-year National Health and Medical Research Council Partnership Grant GNT1161223.	Changes to the context relate to transitioning from RCT to scale-up over time.

* Items and definitions from Hoffmann et al. (16).

### Behavior change technique coding

BCTs targeting child-related behaviors and parent-related behaviors coded for the RCT and scaled-up version of the INFANT intervention are summarized in Tables 3, 4. Examples of intervention content related to the coded BCTs are presented in the Supplementary material. Of the 93 BCTs in the BCTTv1, the RCT included 20 unique BCTs and the scale-up intervention included 28 unique BCTs targeting children's behaviors (Table 3). The RCT and scale-up intervention feature of group sessions included the same 15 BCTs (see Table 3). The only BCT that was coded for the RCT but not for the scale-up related to the provision of tangible tools between sessions, such as balls for children's physical activity (BCT 12.5 adding objects to the environment). There were nine BCTs coded for the scale-up version of INFANT only, and all were coded from the app.

**Table 3:** A comparison of BCTs in the INFANT RCT and scale-up targeting children's feeding practices, nutrition, physical activity and sedentary behaviors.

**BCT number and label**		**RCT**		**Scale-up**		**RCT and Scale-up**
		**Sessions**	**Between-session materials**	**Sessions**	**App**	**Videos**
1.1	Goal setting (behavior)	✓		✓	✓	
1.2	Problem solving	✓		✓	✓	
1.5	Review behavior goal(s)	✓		✓		
2.2	Feedback on behavior				✓	
2.3	Self-monitoring of behavior				✓	
3.1	Social support (unspecified)	✓	✓	✓	✓	
3.2	Social support (practical)		✓		✓	
4.1	Instruction on how to perform a behavior	✓	✓	✓	✓	✓
4.2	Information about antecedents				✓	
5.1	Information about health consequences	✓	✓	✓	✓	✓
5.2	Salience of consequences				✓	
5.3	Information about social and environmental consequences	✓	✓	✓	✓	
6.1	Demonstration of the behavior	✓		✓	✓	✓
6.2	Social comparison	✓		✓	✓	✓
7.1	Prompts / cues		✓		✓	
8.1	Behavioral practice / rehearsal	✓	✓	✓	✓	
8.2	Behavioral substitution	✓	✓	✓	✓	
8.3	Habit formation				✓	✓
8.6	Generalization of a target behavior				✓	
8.7	Graded tasks				✓	
9.1	Credible source	✓	✓	✓	✓	✓
10.4	Social reward				✓	
10.9	Self-reward				✓	
11.2	Reduce negative emotions		✓		✓	✓
12.1	Restructuring the physical environment	✓	✓	✓	✓	✓
12.2	Restructuring the social environment	✓	✓	✓	✓	
12.5	Adding objects to the environment		✓			
13.1	Identification of self as a role model	✓	✓	✓	✓	
15.1	Verbal persuasion about capability				✓	
	Total number of BCTs	15	14	15	27	8

**Table 4:** A comparison of BCTs in the INFANT RCT and Scale-up targeting parents' own nutrition and physical activity behaviors.

**BCT number and label**		**RCT**		**Scale-up**	
		**Sessions**	**Between-session materials**	**Sessions**	**App**
1.2	Problem solving	✓		✓	
1.5	Review behavior goal(s)	✓		✓	
2.2	Feedback on behavior	✓			✓
2.3	Self-monitoring of behavior	✓	✓		✓
3.1	Social support (unspecified)	✓	✓	✓	✓
3.2	Social support (practical)		✓		✓
4.1	Instruction on how to perform a behavior	✓	✓	✓	✓
5.1	Information about health consequences	✓	✓	✓	✓
5.3	Information about social and environmental consequences		✓		✓
5.6	Information about emotional consequences		✓		✓
6.1	Demonstration of the behavior				✓
7.1	Prompts / cues		✓		✓
8.1	Behavioral practice / rehearsal		✓		✓
8.2	Behavioral substitution				✓
8.3	Habit formation		✓		✓
8.7	Graded tasks				✓
9.1	Credible source	✓	✓	✓	✓
10.4	Social reward				✓
12.1	Restructuring the physical environment	✓	✓	✓	✓
12.5	Adding objects to the environment		✓		
13.1	Identification of self as role model	✓	✓	✓	✓
13.2	Framing / reframing		✓		
	Total number of BCTs	10	15	8	18

For the parent-focused behaviors, 18 BCTs in the RCT intervention and 20 BCTs in the scale-up intervention were identified (Table 4). The RCT and scale-up intervention feature of group sessions included the same nine BCTs, with two additional BCTs in the RCT sessions (Table 4). The RCT version of INFANT included activities for parents to monitor and receive feedback on their own behaviors e.g., tracking their physical activity and assessing their diets (BCT 2.3 Self-monitoring of behavior and 2.2 Feedback on behavior). These activities were removed from the scale-up session when the session duration was reduced, and were instead included in the scale-up app. Same as for the child-related behaviors, BCT 12.5 (adding objects to the environment) was coded for the RCT but not for the scale-up, based on the provision of tangible tools between sessions, such as pedometers for monitoring parents' steps. The app incorporated four BCTs unique to the scale-up intervention.

### Rationale for BCT changes between RCT and scale-up

The differences in BCTs coded for the RCT compared to the scaled-up version of INFANT related to modifications made for intervention scalability. For example, the BCT 12.5 (adding objects to the environment) that was coded for the tangible tools given out to participants during the RCT (e.g., balls, fridge magnets, a pedometer for parents). This BCT was not present for the scale-up as these tools were not given out during the scale-up due to funding and logistics of delivering to sites. Notably, there were more unique BCTs present in the scale-up app, primarily due to the app's scope to include more activities and topics (including milk feeding content) which could be coded from explicitly stated written materials.

### BCT coder agreement

BCT inter-coder reliability, measured using PABAK, ranged from 0.76 to 0.89 for child- and parent-related behaviors targeted in all the coded RCT intervention materials (substantial to excellent agreement). Inter-coder reliability for identifying BCTs in the scaled-up version of INFANT ranged from substantial agreement for both child-related and parent-related behaviors targeted in the sessions (0.76 and 0.74, respectively), and moderate to fair agreement for child-related and parent-related behaviors targeted in the mobile app (0.44 and 0.40, respectively).

## Discussion

This is the first study to examine the components (target behaviors, delivery features and BCTs) of INFANT, an early life nutrition and movement behavioral intervention from RCT to scale-up. We found few published research studies that presented BCTs of scaled-up behavior change interventions in other disciplines. For example, one study described using BCTs to inform implementation strategies (43) and another examined BCTs in publicly available apps (44). Yet, none explored changes from RCT to scale-up. This study offers an important and unique contribution to the literature, unpacking what and why intervention components, including BCTs, were altered from RCT to scale-up of the INFANT intervention.

Our results highlight that the scaled-up version of the INFANT intervention stayed true to the initial purpose to promote healthy nutrition and movement behaviors in early childhood. The target population and target behaviors were expanded, and the delivery features were adapted for scale-up. The BCT mapping showed that the intervention was largely using the same techniques to change behaviors. The main changes seen in BCTs identified for the RCT vs. scale-up corresponded to modifications made for scalability and in response to temporal changes, such as the enhanced technology available, with these two iterations occurring more than a decade apart. With the exception of new content around milk feeding (breastfeeding, formula feeding and mixed feeding), we utilized app capabilities (push notifications, quizzes providing personal feedback, parent forum) to reinforce messages received in the intervention and the app enabled increased opportunity for BCT inclusions. Unpacking the intervention components lays the foundation for understanding the implementation-effectiveness outcomes as the scale-up of INFANT progresses, as well as exploring the reasons for the potential scale-up “penalty” when moving from RCT to scale-up.

In comparing the intervention components of the INFANT RCT and scaled-up versions, it is important to highlight that the scale-up is currently in progress and therefore the reported data is for the planned, rather than actual, intervention implemented. Scaled-up interventions are much more likely to be modified and adapted than an RCT given that scale-up implementation is led by local delivery organizations and applied to local contexts (45), whereas an RCT is led by interventionists and conducted in a controlled manner. In addition to the planned local adjustments documented in this study (e.g., involvement of varied practitioners as facilitators, varied settings for delivering sessions), there are likely to be unplanned adjustments to the intervention and its delivery according to local contexts, these may include modifications to the sessions to suit local populations (31, 45). The use of an app in the scale-up does offer technological advantages for easily capturing engagement data and ensuring consistency in information to parents, however the group session content and delivery are more open to local modifications. We aim to capture and explore rationale for local adaptations and modifications in the evaluation of INFANT scale-up through documented local implementation plans collected at baseline (prior to implementation) and yearly thereafter, surveys with implementers (collected at baseline, 12 and 24 months), and semi-structured interviews with a purposeful sample of implementers at 12 and 24 months (low/high adaptions, implementers/non-implementers) (32).

Little is known about the associations between intervention components, including BCTs, and outcomes, and assessing this is challenging (46). Previous systematic reviews have identified BCTs associated with effective interventions related to childhood obesity prevention (21–23), yet BCTs identified were inconsistent between reviews. Matvienko-Sikar et al. (22) identified eight BCTs using BCTTv1 associated with effective health professional-delivered infant feeding obesity prevention interventions (1.2 problem solving, 1.5 review behavior goal(s), 2.2 feedback on behavior, 2.7 feedback on outcome(s) of behavior, 3.1 social support (unspecified), 4.1 instruction on how to perform a behavior, 5.1 information about health consequences and 6.1 demonstration of the behavior), of which seven of the eight identified are used in the INFANT RCT and scale-up intervention. However, contrastingly, Anselma et al. (23) found no major differences between identified BCTs in effective vs. non-effective interventions. It should be noted that such reviews have often relied on arbitrary cut-offs for effectiveness and further exploration of effectiveness of BCTs is required using a large sample of interventions; the TOPCHILD Collaboration is seeking to address this gap (20).

There is limited evidence regarding an ideal number of unique BCTs to support behavior change and scarce evidence about the optimal frequency or “dose” of BCTs. It is plausible that a greater number of unique BCTs and repetition throughout an intervention could see greater behavioral outcomes. A systematic review of internet-based behavior change interventions found that the greater number of BCTs used correlated to larger intervention effects (47). Yet, a 2019 publication by JaKa et al. (48) found that the number of unique BCTs used in an obesity prevention intervention was not associated with change in child BMI percentile. No studies were identified that investigate the optimal repetition or dose of BCTs. It was beyond the scope of the current project to capture the frequency of each identified BCT. Future research using the app in the INFANT scale-up could allow for future exploration of BCT dose by using technology to capture metrics of participant engagement with different components and BCTs, and the association with behavioral outcomes. Other intervention factors relating to dose may also have importance related to behavioral outcomes, for example length of sessions or contact-time (48, 49).

### Strengths and limitations

The key strengths of this study include best practice methods and reporting for BCT coding (33), including use of standardized coding processes, independent coders, and reporting of coder agreement. Also, we used established frameworks to categorize intervention components (16, 17), and had strong involvement from the RCT and scale-up interventionists. Commonly, BCT assessment relies on often poorly described intervention characteristics in published materials which, in turn, impacts the accuracy of coding (33, 50). This study addressed this issue through access to unpublished material and undertaking a novel validation process with interventionists.

Limitations of this study include coding BCT presence but not frequency. Coding BCT presence is common practice when coding BCTs, however without understanding the frequency of BCTs in an intervention it is not possible to understand dose nor infer relationships with outcomes. Another limitation is the moderate agreement between coders when BCT coding the scale-up intervention app. Moderate inter-coder agreement was seen by others coding childhood obesity prevention and treatment interventions (51), highlighting the complexities of the deductive coding process. In this study, the moderate agreement scores were likely in-part due to the approach undertaken for coding the app; due to the volume of content in the app, the coders randomly selected sections from all features of the app opposed to coding line-by-line and therefore resulted in differing independent results. However, all coders had undertaken the BCT training, used the same supporting resources and followed the same process. All coding was based on intervention materials, and there were thorough coding consensus and validation meetings with an experienced BCCTv1 coder external to the intervention and interventionists (including two interventionists who led the original RCT and remain involved in leading the scale-up) that ensured rigorous debate and accurate resolution of discrepancies.

### Implications for future research

While there has been an increase in family-based intervention trials for early childhood obesity prevention and a rise in the unpacking of intervention components to explore “active” elements, there has been no investigations of the intervention components once scaled-up. This is partly due to there being few early childhood obesity prevention trials being scaled-up. Acknowledging the many factors that contribute to whether an intervention progresses to scale-up (52), we recommend that trials that do scale up also assess the discrete intervention components. This study offers a template for other interventions in undertaking such work. Future scale-up of effective early childhood obesity prevention interventions and increased research into unpacking the components of interventions, will allow further examination into the scale-up penalty. An important step for the INFANT research will be to assess the effectiveness of the intervention at scale [currently underway (32)] and compare this with the RCT. Having the intervention components explicitly presented in this study, researchers will be able to consider factors contributing to effective outcomes. Standardized reporting of intervention delivery features with sufficient details, publishing intervention protocols and materials or making them accessible via research groups are essential for accurate understanding of interventions and identification of BCTs (53).

## Conclusion

This unique study examined the components (target behaviors, delivery features and BCTs) of INFANT, an early life nutrition and movement behavioral intervention from RCT to scale-up. Findings show that while the scaled-up version of the INFANT intervention had modifications to target behaviors and delivery features for scalability, the techniques identified to change behaviors were largely consistent. Other behavior change interventions enacting scale-up, particularly for early childhood obesity prevention, should consider undertaking similar research to increase understanding and transparency of what and why changes were made to a scaled-up intervention and the active ingredients for changing behavior. Scale-up of early childhood nutrition and movement behavioral interventions is critical for achieving population-level health benefits; this work presents INFANT as an example and lays the foundation for investigating scale-up implementation-effectiveness outcomes.

## Data availability statement

The raw data supporting the conclusions of this article will be made available by the authors, without undue reservation.

## Ethics statement

Ethical review and approval was not required for this study in accordance with the local legislation and institutional requirements. Written informed consent from the program designers was not required to participate in this study in accordance with the national legislation and the institutional requirements.

## Author contributions

SM, PL, RL, KH, ED-W, and KC conceptualized this study. SM administered the project. SM, PL, RL, KH, ED-W, KC, and BJ designed the methodology. SM, BJ, and KF coded the trial, conducted the formal analysis, and presented the data. SM wrote the original draft with input from BJ and KF. All authors contributed to reviewing and editing the manuscript and approved the final version.
