# Supplementary material for: Mapping intervention components from a randomized controlled trial to scale-up of an early life nutrition and movement intervention: The INFANT program
Source: Front Public Health. 2023 Jan 13;10:1026856. doi: 10.3389/fpubh.2022.1026856 (PMC9880042; doi:10.3389/fpubh.2022.1026856)
Supplement: Supplementary file 1 [file Table_1.DOCX]

**Supplementary Material**

**Table 1. Intervention materials coded for the INFANT RCT and scale-up intervention**

| **Intervention material** | **RCT** | **Scale-up** |
| --- | --- | --- |
| Facilitator session guide |  | (Updated version) |
| Session videos |  | (Condensed length) |
| Session handouts |  | (Updated versions) |
| Newsletters (between sessions) |  | Not applicable |
| Additional objects for participants (e.g., ball, fridge magnet with key messages, pedometer) |  | Not applicable |
| My Baby Now phone app | Not applicable |  |
| Published research articles |  |  |

**Table 2. Examples of intervention content and identified BCTs in the INFANT RCT and scale-up targeting children’s feeding practices, nutrition, physical activity and sedentary behaviors**

|  | **RCT** | | **Scale-up** | | **RCT and Scale-up** |
| --- | --- | --- | --- | --- | --- |
| **Behavior Change Technique (from BCTTv1^1^ )** | **Sessions** | **Between-session materials (Handouts / Newsletters /Tangible tools)** | **Sessions** | **App** | **Videos** |
| 1.1 Goal setting (behaviour) | Discussion with interventionists – facilitators were encouraged to ask parents to select ‘one thing’ [goal] to work on between session. Intention was prompting reflection, troubleshooting barriers, for parents to consider their goals. |  | Example from facilitator manual – “Ask participants to share one thing they have learnt or something they might try to do as a result of the session.” | Example from ‘My breastfeeding goals’ activity: “I plan to breastfeed for <select from options>” |  |
| 1.2 Problem solving | Example from main results paper – “Social cognitive theory guided program development, incorporating a range of delivery modes and educational strategies including group discussion and peer support, as well as exploration of facilitators and barriers to uptake of key messages.”  Example from facilitator manual – “Estimate how much time your child sends doing activity. Are you and your child meeting these goals? What is making it hard to reach these goals? Discuss suggestions to overcome this” |  | Example from facilitator manual – “The aim of the facilitated discussion is to provide opportunity for parents to share and learn from each other, particularly around practical ways to address common barriers.” | Example from ‘Feeding’ topics -- Strategies provided to common problems (e.g., breastfeeding) and advice provided for strategies to try. |  |
| 1.5 Review behaviour goal(s) | From facilitator manual – “summary of what’s been learnt” at the start of each session, and review of each key message.  Discussion with interventionists – intended as an opportunity to reflect on goals and discuss as a group. |  | Example from facilitator manual – “Session Check-in: Share one key thing they have learnt/tried/done differently as a result of attending INFANT sessions.” |  |  |
| 2.2 Feedback on behaviour |  |  |  | Example from activities –  Participant answers questions about behaviour (e.g., How often do you offer your baby vegetables?”) and responses are feedback tailored to the participant. This includes both praise and tips/links to information to perform the behaviour. |  |
| 2.3 Self-monitoring of behaviour |  |  |  | Example from vegetable tracker activity – “To get help tracking the number of times you’ve exposed your little one to a vegetable, try our vegetable tracker.” |  |
| 3.1 Social support (unspecified) | Example from protocol paper – “Based on an anticipatory guidance framework the intervention will incorporate a range of modes of delivery and educational strategies including brief didactic sessions, use of group discussion and peer support, exploration of perceived barriers, use of visual and written messages, follow-up delivery of messages by text-messaging and mail-outs.” | Example from newsletter –"Remember that showing the DVD to partners, grandparents and carers (even just a section or two!) is a good way of helping others to understand what you believe about healthy eating and physical activity” | Example from facilitator manual --  Ask a health professional for help to find an approach that works for you. | Example from ‘Help and support’ topic --  Range of local support services listed in help. Parenting forum. |  |
| 3.2 Social support (practical) |  | Example from handout -- “YOU’RE A DAD- so what is your role? … Ultimately you and your partner together need to set the family rules around meal times, TV viewing and being active. Support each other in your ideals, stick to the rules you create and role model them yourself if you expect your child to follow suit. They’ll certainly notice if you don’t! |  | Example from ‘For parents’ topic – “Don't be afraid to ask someone to help you with practical jobs, such as washing, shopping, etc.” |  |
| 4.1 Instruction on how to perform a behaviour | Example from facilitator guide – instructions about how to prepare mashed and soft foods for babies. E.g., Fruit and veg make great snacks for babies. Soften hard fruit and veg (like broccoli, carrot and apple) by stemming them. | Example from newsletter – “A great active game: Follow the leader. You can be very imaginative with this game and add more challenging actions as your child grows. Sing “Just like me” as you do this if you like.” | Example from facilitator manual --  Reinforce signs of fullness (closing mouth tightly, turning head away, pushing away spoon). | Example from ‘Solids’ topic – “Try one spoon at a time, one for me one for you. As baby gets older encourage them to feed themselves.” |  |
| 4.2 Information about antecedents |  |  |  | Example from feed, play, sleep topic, crying and settling: “You might find it helpful to go through the below questions to work out the cause of your baby’s crying, which will then allow you to quickly help your baby to settle (and reduce your stress levels!). Could your baby be hungry, upset or tired?...” |  |
| 5.1 Information about health consequences | Example from facilitator manual – “Note that in their handouts we have provided a copy of the “Go for your Life” leaflet “Why no sweet drinks for children”. This provides lots of good talking points about the rationale for limiting sweet drinks. Please refer to this.” The leaflet includes information about health problems i.e., sugary drinks are not necessary and may cause health problems.  Example from facilitator manual – “The idea that we should “Color Every Meal with Fruit and Veg?” – is this achievable? Is it what you think makes for a healthy diet? (use this to explore the notion that eating a wide range of fruit and veg is likely to be the most protective thing we can do for our health).” | Example from handout -- “A range of foods make up a healthy diet however, ‘sometimes foods’ should be limited to no more than 2-3 times a week because they are not essential and displace foods needed for growth and development.”  Example from newsletter --“Avoid using a baby’s bottle for comfort as this can lead to tooth decay.” | Example from facilitator guide – “Reinforce importance of introducing a sippy cup with water at meals and offering water, breastmilk/ formula as the only drinks. [reference to “Drinks other than milk and water --For example, juices (including 100% fruit juice), soft drinks, cordials or sports and energy drinks can:  • damage growing teeth – even before they have come through”] | Example from ‘Play’ topic - “Research shows that babies who watch TV or have other screen time, have:  • shorter attention spans  • altered development of their eye strength and movement” | Example from video - “Breastmilk (or formula) gives all the nutrition needed.” |
| 5.2 Salience of consequences |  |  |  | Example from ‘Family meals’ topic –  Images of young children eating same meal as parent. |  |
| 5.3 Information about social and environmental consequences | Example from facilitator notes - “Playing with your baby is important and good fun.” | Example from handout -- “Action rhymes and songs encourage your child to experience different types of movement. They’re a form of active play but most importantly they’re great fun!” | Example from facilitator manual –  “Family meals and playtimes help your baby learn about communication, social interaction and manners.” | Example from ‘family meals’ topic –  “Feeding your baby the same food as the rest of the family not only saves you time but also encourages your baby to eat the same foods as the family!” |  |
| 6.1 Demonstration of the behaviour | Example from facilitator notes -- “Mash up a banana and an avocado to provide examples of what these foods will look like/taste like.” |  | Example from facilitator manual –  “Discuss/demonstrate alternative positions for tummy time and tummy time tips/strategies for babies who don’t like tummy time” | Example from ‘breastfeeding’ topic --  “These images show what early, active and late signs of hunger look like in a baby.” | Example from video --  Video shows parent feeding baby    Mum sharing about what has worked for her with repeatedly offering vegetables    Mum sharing about what has worked with setting up a mealtime routine and environment |
| 6.2 Social comparison | Facilitator manual –  Each session prompts discussion of key barriers to the program key messages. E.g. “Discussion of points of interest and exploration of key barriers to key messages” Prompt: “What do you think about?: The idea that we should “Color Every Meal with Fruit and Veg?” – is this achievable?”  “The suggestion that it is recommended that babies under 2 should not watch television at all? Had you heard this before? Why is this a recommendation? (taps into the research re babies brain development shared from the Health Report transcripts). Is this going to be hard to do? Is this what other parents you know have done?”  Discussion with interventionists – social comparison during these discussions was intended, with it being an opportunity to compare challenges and solutions to behaviour change with other parents |  | Example from facilitator manual –  “What types of active play are people doing with their baby? Ask participants to demonstrate how they play with their baby.” | Example from ‘Play’ topic --  “Research has shown that babies who regularly practice tummy time in their first 6 months of life (while awake), achieve developmental milestones significantly earlier than those who only do tummy time occasionally in their first 6 months.” | Example from videos – show examples of other parents’ behaviours regarding food provision  Discussion with interventionists – there were several purposes of the videos, one of which was to provide examples for parents to compare and relate themselves to |
| 7.1 Prompts/ cues |  | Example from newsletter--“Also the Key Messages chart that you received during your first INFANT session (with the fridge magnet) is great to refer back to from time to time!”  Magnetized Key Messages Chart titled Eating and Activity for your baby - Getting it right from the start [i.e. Infant program session messages] |  | Example from ‘About MBN’ topic --  Parents receive 3 personalized push notifications a week tailored to their baby’s age, stage of development and feeding mode (breast or formula feeding). |  |
| 8.1 Behavioral practice / rehearsal | Example from facilitator manual – Session 3 activity “How to roll a ball with your baby - In this session we have given all the families a large ball. Given this we thought a fun activity would be to ask them to pop their babies on the floor in front of them and to roll the ball to their bubs. This should be fun and will take a good 10 minutes.” | Example from newsletter--  “Parents are great teachers. Your child needs you to show them how to catch and throw a ball, hop and skip! They need lots of practice with this and you make a great playmate.” | Example from session –  “Keep offering foods your baby initially rejects, they can take up to 15 tries of a food before they learn to like it.” | Example from ‘Feeding’ topic -- How can I get my baby to drink from a cup? Practice, practice and more practice! |  |
| 8.2 Behavioral substitution | Example from facilitator manual - “Make sure your baby is hungry – offer food and water; don’t bribe or coerce/leave for a small amount of time/ don’t get into the habit of providing “backups”. Sessions’ discussion activities prompted parents to consider barriers and solutions, including substituting behaviors. | Example from handout – “Offer a piece of fruit rather than juice, cow’s milk rather than toddler milks.” | Example from session --  “Even if you think your child won’t eat them – offer veg and fruit as snacks instead of biscuits or other processed food.” | Example from ‘Play’ topic --  “Playing and exploring is much better for their development than offering screens to keep them entertained.” |  |
| 8.3 Habit formation |  |  |  | Example from ‘Family meals’ topic -- “Parents are encouraged to create a mealtime routine to follow every feeding occasion“ | Example from video -  “Not adding salt or sugar is a good habit for you and Phil as well, and is a really good habit to get into with her as well.” |
| 8.6 Generalization of a target behaviour |  |  |  | Example from ‘Play’ topic – “When first starting out, feel free to sit on the floor and just watch your baby explore. Eventually, you will be about to work or relax nearby while they play independently.” |  |
| 8.7 Graded tasks |  |  |  | Example from ‘Solids’ topic – This section includes step-by-step graded tasks to transition from bottle to cup. |  |
| 9.1 Credible source | Sessions facilitated by a dietitian | Session handouts from various reputable sources such as the Australian Government.  Discussion with interventionists – intention was to highlight materials were from reputable sources to build credibility. | Example from facilitator manual --“INFANT is an evidenced based program developed by experts at Deakin University’s Institute for Physical Activity and Nutrition.” | Example from ‘About MBN’ topic – “App content developed by experts using evidence-based research.” | Example from video --Celebrity mum, dietitian feature in the video/s |
| 10.4 Social reward |  |  |  | Example from quizzes --  “Fantastic! You're meeting the recommendation of no screen time for children under the age of 2 - well done!” |  |
| 10.9 Self-reward |  |  |  | Example from ‘Feeding’ topic – “Be pleased! Getting your baby to give up the bottle can be a challenging process but you should also take a moment to feel pleased! “ |  |
| 11.2 Reduce negative emotions |  | Example from handout – “We’ll also talk about the best ways of feeding – so that no-one gets stressed!” |  | Example from ‘Solids’ topic – “Keep your stress levels down by having meal time routine and not letting meal times drag on for ages.” | Example from video -- "you might want her to like pumpkin for example… if she doesn't like and you start [making a fuss] they pick up on it… the more fuss you make, the more fuss they make. So a really important skill with feeding babies is not to get emotional, not to show a response. offer her some food, if she doesn't have it... take it away and say nothing, do nothing". |
| 12.1 Restructuring the physical | Example from facilitator manual -- Encourage baby’s movement and provide a safe environment for them to play and explore. | Example from handout – Helpful strategies include: have healthy food available at home | Example from facilitator manual -- Encourage baby’s movement and provide a safe environment for them to play and explore. | Example from ‘Family meals’ topic – “Set a good example at meal times and put your phone away when you’re eating and playing together.” | Example from video – Video discusses restructuring the pantry and not having sometimes food in the home |
| 12.2 Restructuring the social environment | Example from facilitator manual -- “Grandparents and Carers – helping parents to get it Right from the Start! We have produced two resources dealing with the issue of grandparents and carers – one for the parent and one to pass on to grandparent or carer. Use this resource to generate a discussion re how carers may be feeling about “new” ways of doing things and about how they can help” | Example from handout –  Ensure all carers are consistent with feeding rules for your child | Example from facilitator manual --  “Discuss tips for communicating with other carers about food, screen time and active play” | Example from ‘Breastfeeding’ topic –  Surround yourself with people that support your decision to breastfeed. |  |
| 12.5 Adding objects to the environment |  | Various tangible tools provided to parents such as balls to play with, active-play storybook.  Example from newsletters - “Remember to take a look at the “Just Add Fruit and Veg” cards that we gave you in Session 3. These cards have lots of good ideas that will help you to colour every meal with fruit and veg. We also gave you two charts that talked all about buying fruits and veg when they are in season.” |  |  |  |
| 13.1 Identification of self as role model | Example from facilitator manual – “Babies model themselves on you – if you eat fruit and veg and enjoy them – they will too.”  “Parents are the best teachers for physical activity - if you are active, your kids will be active too” | Example from handout – “Do things together, you are your child’s role model, and they just love to spend time with you.” | Example from facilitator manual –  “Set a good example at mealtimes and put your phone away when you’re eating and playing together.” | Example from ‘Solids’ topic – “One of the best ways to encourage your baby to eat healthy foods is by eating and enjoying them yourself!” |  |
| 15.1 Verbal persuasion about capability |  |  |  | Example from ‘Milk Supply Quiz’– “There is no need to feel like a failure or give up breastfeeding - there are many things you can do to make more [hyperlink to milk supply section]” |  |

**Table 3. Examples of intervention content and identified BCTs in the INFANT RCT and scale-up targeting parents’ nutrition, physical activity, sedentary behaviors and wellbeing**

|  | **INFANT RCT** | | **INFANT Scale-up** | |
| --- | --- | --- | --- | --- |
| **Behavior Change Technique (from BCTTv1^1^ )** | **Sessions** | **Between-session materials (Handouts / Newsletters /Tangible tools)** | **Sessions** | **App** |
| 1.2 Problem solving | Example from facilitator manual -- “Explore barriers to adding fruit and veg to their own diets – did they use any of the suggestions from the last leaflets – adding fruit and veg at lunch?” |  | Example from facilitator manual – “What are some of the barriers to eating more vegetables as a family? Invite ideas for addressing barriers. Discuss tips for getting enough fruit & vegetables.” |  |
| 1.5 Review behaviour goal(s) | From facilitator manual – “summary of what’s been learnt” at the start of each session, and review of each key message.  Discussion with interventionists – intended as an opportunity to reflect on goals and discuss as a group. |  | From facilitator manual – “summary of what’s been learnt” at the start of each session, and review of each key message.  Discussion with interventionists – intended as an opportunity to reflect on goals and discuss as a group. |  |
| 2.2 Feedback on behaviour | Example from main outcome paper -- A range of cognitive feedback activities were employed to promote parental examination of personal eating, physical activity and sedentary behaviors. Emphasis on these behaviors focused on the importance of personal health |  |  | Example from activities/quizzes - Participant self-rates eating habits, quiz-generates personalized feedback. |
| 2.3 Self-monitoring of behaviour | Example from facilitator guide – “Activity -Ask parents to think about their own food choices at home, reminding them that role modelling plays a very large part in their child’s development and early food and eating patterns.  We would like them to record their intake of fruit, vegetables, water and “sometimes” foods over the last two days on the food record sheets provided (show these and an example.)  Also give to them pictures of equivalent amounts as to what a “serve” would be (go thru handout thoroughly) to help them in recording their intake. They do not need to record everything they eat – only the things that fall into these groups. Please give them time to do this on their own, and then discuss their experiences in the larger group.” | Example from newsletter --  “You were given a pedometer – this may still be in its box! The idea of using a pedometer is that it gives you some feedback about how many steps you take each day. The only thing you need to remember is to attach it each morning (just above your hip) and to walk as often as you can throughout the day.” |  | Example from ‘Play’ topic --  You may like to keep a record of your time spent in ‘screen time’ that is not work related for the next week. It can be interesting to take a look at your own behaviours. |
| 3.1 Social support (unspecified) | Example from protocol paper – “..the intervention will incorporate a range of modes of delivery and educational strategies including brief didactic sessions, use of group discussion and peer support.” | Example from handout --  “For much more information about the benefits of exercise go to your folder and take a look at the leaflet “An active way to better health”. | Example from facilitator manual – “Ask about baby friendly physical activity options people know about (yoga, forming a pram walking group, good paths for pram walking etc)” | Example from ‘Help and support’ topic -- Range of local support services listed in help (i.e.  getting active for new parents). |
| 3.2 Social support (practical) |  | Example from handout --  “Ask a friend or family member to join you – physical activity can also be an enjoyable social occasion.” |  | Example from ‘For parents’ topic -- Put aside time each week where your partner or friend takes care of dinner so you can get out and do something active (such as yoga/walking etc.). |
| 4.1 Instruction on how to perform a behaviour | Example from facilitator manual – group discussion of how to add more veg -“Take the time to discuss favourite ways of including vegetables in their own diets. Carolyn has asked mums the following questions to good effect: “what’s your favourite vegetable and how do you cook it?” “what veg would you like to try but don’t know how to cook?”. These seem like good questions for prompting discussion and sharing knowledge and skills” | Example from newsletter-  “About Keeping Physically Active. Tips provided on how to plan and prepare healthy meals and keep active.” | Example from facilitator manual –“Any thoughts on how you can build more physical activity into your own routine and as a family? Discuss tips for moving more.” | Example from ‘Healthy eating’ topic – “Tips for eating more vegetables in your own diet!” |
| 5.1 Information about health consequences | Example from facilitator manual “Emphasis on the importance of exercise – particularly walking.” | Example from handout --  “Just 30 minutes every day can increase cardiovascular fitness, strengthen bones, reduce excess body fat and boost muscle power and endurance” | Example from facilitator manual – “Discuss benefits of pram walking for their own wellbeing.” | Example from ‘For parents’ topic –“Despite all the marketing claims, these drinks tend to only give you a quick boost, usually followed by a slump of feeling even more tired.” |
| 5.3 Information about social and environmental consequences |  | Example from newsletter--  “Remember that getting your own health in order has lots of benefits – it looks after you (and you are the most important person in your baby’s life!) and it also means your baby will grow up learning how to take good care of themselves.” |  | Example from ‘Healthy eating’ topic – “Healthy eating can save you both valuable time and money!” |
| 5.6 Information about emotional consequences |  | Example from handout--  “Feel invigorated and happy after each walk – enjoy these opportunities to relax.” |  | Example from ‘Healthy eating’ topic – A healthy diet will boost your wellbeing and keep you energized, which can promote positive mental health (mood). |
| 6.1 Demonstration of the behaviour |  |  |  | Example from ‘For parents’ topic – Pictures demonstrating (and instructions) how to perform pelvic floor exercises |
| 7.1 Prompts/ cues |  | Example from newsletter--  “Also the Key Messages chart that you received during your first INFANT session (with the fridge magnet) is great to refer back to from time to time!”  Lunch boxes provided with "snack on fruit and veg" sticker |  | Parents receive personalized push notifications, of which some target parents’ behaviours e.g., “Trying to eat more healthily? The foods you keep in the house will influence what you eat and the eating habits that [baby name] develops. More tips here.” |
| 8.1 Behavioral practice / rehearsal |  | Example from handout --  Schedule a regular family walk – this is not only a great way to establish a good healthy family routine, but allows you to spend quality time together with your partner and your baby. |  | Example from ‘Breastfeeding’ topic -- Prompts mums to remember to drink water when breastfeeding "keep a reusable water bottle by your side to remind you to drink it" |
| 8.2 Behavioral substitution |  |  |  | Example from ‘Healthy eating’ topic – Try spreading avocado on toast (or even a little drizzle of olive oil) instead of butter. |
| 8.3 Habit formation |  | Example from handout and session messages -- "Develop the habit of ‘going for a walk’ every day.”  “Include walks, park visits and other active play in your family routine.” |  | Example from breastfeeding section --prompts mums to remember to drink water when breastfeeding "keep a reusable water bottle by your side to remind you to drink it"  Discussion with interventionists – content throughout refers to repeated behaviours intended to form habits. |
| 8.7 Graded tasks |  |  |  | Example from ‘For parents’ topic -- Start off slowly (exercise) and then gradually increase how hard you go and how long for as long as you feel more able. |
| 9.1 Credible source | The intervention will be delivered by a dietitian and is comprised of six sessions delivered at three-month intervals during the regular meeting time of the first-time parents' group | Session handouts from various reputable sources such as the Australian Government.  Discussion with interventionists – intention was to highlight materials were from reputable sources to build credibility. | Example from facilitator manual --“INFANT is an evidenced based program developed by experts at Deakin University’s Institute for Physical Activity and Nutrition.” | Example from ‘About MBN’ topic – “App content developed by experts using evidence-based research.” |
| 10.4 Social reward |  |  |  | Example from ‘How healthy is your diet’ activity – General encouragement, e.g.  “Keep up the great work!” |
| 12.1 Restructuring the physical | Example from facilitator manual “If you make a decision to eat more fruit and veg then you’ll have more of it in the house – which means these terrific foods will be there when you want to offer them to your baby.” | Example from program summary handout --“Strategies for reducing ‘sometimes foods’ include: not buying them often as you are less likely to eat them if they aren’t in your cupboard; buying only small amounts; leading by example; and not shopping when hungry.” | Example from parent handout referred to during the sessions --  “Have chopped up veg and fruit handy for easy snacks for you”. | Example from ‘Play’ topic –  Keep TV’s and computers out of bedrooms. |
| 12.5 Adding objects to the environment |  | Parents given pedometers to self-monitor their physical activity |  |  |
| 13.1 Identification of self as role model | Example from facilitator manual – “Ask parents to think about their own food choices at home, reminding them that role modelling plays a very large part in their child’s development and early food and eating patterns.” | Example from handout --  “Remember that children are more likely to be active over their life if the family does regular physical activity together. This is a great reason for you to be active too! “ | Example from facilitator manual -- “Remind parents when it comes to feeding your baby- you are your baby’s most important role model. “ | Example from ‘Solids’ topic --  Because your baby is always watching what you do and will see you eating fruit regularly, they will want to eat it too, which sets them up for a healthy start to life! |
| 13.2 Framing / reframing |  | Example from handout --  But now that you’re a mum, looking after yourself is more important than ever. We all know that physical activity is important, but it often falls into a category we call ‘me time’ ‘Me time’ can be elusive, and as guilt ridden as that bar of chocolate. But it’s essential that you take the time to make physical activity a priority in your life. Ultimately, if you look after yourself, your baby and family will benefit from having a happier and healthier mum. So reframe ‘me time’ to ‘I’m doing this for myself and my family’. |  |  |

^1^ Behavior Change Techniques coded using the BCTTv1 from Michie et al. 2013
